# Effect of AGM and Fetal Liver-Derived Stromal Cell Lines on Globin Expression in Adult Baboon (*P. anubis*) Bone Marrow-Derived Erythroid Progenitors

**DOI:** 10.1371/journal.pone.0036846

**Published:** 2012-05-31

**Authors:** Donald Lavelle, Kestutis Vaitkus, Maria Armila Ruiz, Vinzon Ibanez, Tatiana Kouznetsova, Yogen Saunthararajah, Nadim Mahmud, Joseph DeSimone

**Affiliations:** 1 Department of Medicine, University of Illinois at Chicago, Chicago, Illinois, United States of America; 2 Jesse Brown VA Medical Center, Chicago, Illinois, United States of America; 3 Hematologic Malignancies and Blood Disorders, Cleveland Clinic, Cleveland, Ohio, United States of America; French Blood Institute, France

## Abstract

This study was performed to investigate the hypothesis that the erythroid micro-environment plays a role in regulation of globin gene expression during adult erythroid differentiation. Adult baboon bone marrow and human cord blood CD34+ progenitors were grown in methylcellulose, liquid media, and in co-culture with stromal cell lines derived from different developmental stages in identical media supporting erythroid differentiation to examine the effect of the micro-environment on globin gene expression. Adult progenitors express high levels of γ-globin in liquid and methylcellulose media but low, physiological levels in stromal cell co-cultures. In contrast, γ-globin expression remained high in cord blood progenitors in stromal cell line co-cultures. Differences in γ-globin gene expression between adult progenitors in stromal cell line co-cultures and liquid media required cell-cell contact and were associated with differences in rate of differentiation and γ-globin promoter DNA methylation. We conclude that γ-globin expression in adult-derived erythroid cells can be influenced by the micro-environment, suggesting new potential targets for HbF induction.

## Introduction

Developmental changes in expression of the five individual β-like globin genes (globin gene switching) are coordinated with shifts in the site of erythropoiesis from yolk sac to liver to bone marrow during development [1]. Transplantation experiments [2], [3] and studies of cell hybrids formed between murine erythroleukemic cells and human fetal erythroid cells [4] showed that developmental globin switching was controlled by a cell-intrinsic developmental clock and not influenced by extrinsic factors.

In contrast, the capability of adult erythroid progenitor cells to express increased levels of fetal hemoglobin (HbF) when cultured in vitro is well established [5] indicating that cell extrinsic factors present in the environment can alter the globin gene expression program during adult erythroid differentiation. The ability of adult-derived BFUe to express increased levels of HbF in culture is highly influenced by the presence of components in culture medium such as fetal bovine serum [6], [7] and high levels of growth factors including SCF [8], [9]. The ability of high levels of erythropoietin [10], SCF [11] and pharmacological agents [12], [13] to increase HbF levels in vivo further demonstrates that the globin gene expression can be modulated in vivo and that the effect of extrinsic factors on the globin gene expression program is not limited to in vitro culture conditions.

Stromal cells, extracellular matrix proteins, macrophages, and the presence of growth factors within the erythroid niche micro-environment are known to affect the differentiation and survival of erythroid progenitor cells [14]–[16]. Previous studies in ES cells and iPS cells have shown that co-culture with the stromal cells can alter globin gene expression [17]–[19]. Additional studies showing that human cord blood-derived CD34+ progenitors synthesized high levels of HbF when cultured in vitro, but switched to predominately adult hemoglobin (HbA) expression when transplanted into nonobese diabetic, severe combined immunodeficient (NOD/SCID) mice suggested that the differentiating cord blood-derived erythroid cells may have been repropgrammed from HbF to HbA expression by the adult hematopoietic microenvironment [20]. Taken together, these results suggest the hypothesis that, in addition to affecting terminal differentiation and survival, stromal cells within the erythroid niche micro-environment may also influence globin gene expression in primary CD34+ erythroid progenitors. To test this hypothesis, we analyzed the effect of three murine stromal cell lines derived from the aortic-gonadol-mesonephros (AGM) region of the early embryo, fetal liver, and adult bone marrow on globin gene expression during erythroid differentiation of CD34+ baboon adult bone marrow and human cord blood-derived progenitors. The baboon was used as a source of CD34+ bone marrow cells for these studies and has been considered an excellent animal model for studies of globin gene regulation because the structure of the β-globin gene complex and developmental pattern of expression are similar between baboon and man, and expression of the γ-globin gene as a true fetal stage gene is observed only in simian primates.

## Materials and Methods

### Ethics Statement

All procedures involving baboons were approved by the Animal Care Committee of the University of Illinois at Chicago. Human cord blood was obtained from the New York Blood Center (New York, NY) according to Institutional Review Board guidelines. Protocols for isolation and culture of CD34+ cells from human cord blood were approved by the IRB of the University of Illinois at Chicago.

### Cell Lines

The AFT024 fetal liver [21] and OP9 bone marrow-derived [22] stromal cell lines were obtained from ATCC. The U26B1 AGM-derived cell line was obtained from the laboratory of Dr. Elaine Dzierzak of Erasmus University Medical Center [23]. For co-cultures, AFT024 and U26B1 cells were grown as monolayers at 32°C in T25 flasks until cells were approximately 90% confluent. Cultures were shifted to 37°C the day before CD34+ cell isolation.

### Baboons

Baboons were housed at the University of Illinois at Chicago Biologic Resources Laboratory (UIC BRL) under conditions that meet the Association for Assessment and Accreditation of Laboratory Animal Care (AAALAC) standards. The University of Illinois at Chicago is committed to the judicious, humane use of animals in research and teaching and is also committed to maintaining full accreditation by the AAALAC. In accordance with this commitment, all measures are taken to socially house nonhuman primates in pairs or groups in accordance with Section 3.81(a) of the Animal Welfare Standards, when stable pairs can be established or unless otherwise exempt due to veterinary or scientific reasons in facilities that meet AAALAC standards for accreditation. Nonhuman primates receive a variety of foodstuffs in addition to their daily diet of primate biscuits which may include seasonal fruits and vegetables, Softies, and human food treats. They also receive a foraging mixture consisting of a variety of seeds, dried fruit, and dry dog food several times each week, and several times each month receive a novel food enrichment item which may include seasonal fruits (green onions, radishes, melons, oranges, holiday candies, etc.) and treats (frozen juice popsicles popcorn balls etc.). An adequate water supply is continuously available. Baboon bone marrow aspirates were obtained according to a protocol approved by the Animal Care Committee of the University of Illinois at Chicago. Bone marrow aspirations were performed from the hips of animals under ketamine/xylazine anesthesia (10 mg/kg; 1 mg/kg). Prior to bone marrow sampling, buprenex (0.01 mg/kg IM) was given and later in the afternoon a second dose of buprenex (0.01 mg/kg IM) was administered to alleviate potential pain and suffering.

### Isolation and Culture of Bone Marrow and Cord Blood-Derived CD34+ Cells

CD34+ cells were isolated from baboon bone marrow and human cord blood by immunomagnetic column purification as previously described [24], [25]. Mononuclear cells were purified from baboon BM aspirates (30–40 ml) by sedimentation in Percoll gradients. Purification of CD34+ cells was performed by immunomagnetic column chromatography using the anti-CD34 12.8 monoclonal antibody (a gift of Dr. Robert Andrews) as the primary reagent and immunomagnetic rat anti-mouse IgM microbeads (Miltenyi Biotech) as the secondary reagent. CD34+ subpopulations differing in CD36 expression were purified by FACS using mouse anti-human CD36 FITC-conjugated antibody (Clone Beckman Coulter FA6.152) as previously described [26]. For purification of CD34+ cells from human cord blood, mononuclear cells were isolated by centrifugation on Ficoll-Paque (Amersham Bioscience) followed by purification of CD34+ cells using a MACS CD34 progenitor isolation kit (Miltenyi Biotech) were purified from human cord blood and human cord blood.

Isolated CD34+ cells were cultured in liquid media, methylcellulose media, and in co-culture with stromal cell lines in Iscove’s media containing 30% fetal bovine serum (FBS), 2 U/ml EPO, 200 ng/ml SCF, and 1 µM dexamethasone. For co-cultures, media was removed from stromal cell line monolayers and CD34+ bone marrow cells (5–10×10^4^ cells/ml) added. Fresh media containing FBS and cytokines was added on days 5 and 10.

Transwell cultures were performed in 6 well plates using transwell permeable supports with 0.4 µm polyester membranes (Costar). AFT024 or U26B1 cells were grown to confluency at 32°C in DMEM containing 10% FBS in 6 well plates. Media was removed and replaced with 2 ml Iscove’s media containing 30% FBS, 2 U/ml EPO, 200 ng/ml SCF, and 1 µM dexamethasone. CD34+ bone marrow progenitors were added within the transwell insert in 0.5 ml media. On days 5 and 10, 1.0 ml of media was removed from the bottom portion of the transwell and replaced with fresh media.

Conditioned media (CM) was prepared by growing AFT024 or U26B1 cells at 32°C in appropriate growth medium until confluent. Growth medium was then removed, replaced with Iscove’s Media containing 30% FBS and cells incubated at 37°C for three to four days. CM was removed, clarified by centrifugation, filtered through a 0.22 mM filter, and stored at −20°C. For experiments, CM was thawed and mixed with Iscove’s media containing 30% FBS at a 40∶60 ratio and EPO, SCF, and Dex added prior to use in cultures.

Analysis of colony forming cells (CFUe, BFUe) present in cultures on varying days was performed by plating 2000 viable cells in H4034 Methocult media (Stem Cell Technologies) in duplicate 35 mm tissue culture dishes. Colonies (BFUe, CFUe) were counted using a Zeiss Axiovert 40C inverted microscope.

### Analysis of Globin Expression

Globin chains synthesis in cultured cells was measured by biosynthetic radiolabelling as previously described [27]. Cells (2–5×10^5^) were incubated for twenty four hours in leucine-free α-minimum essential media (Invitrogen) containing 50 µCi/ml L[4,5^−3^H leucine (Perkin Elmer). Following radiolabelling, cells were washed three times in cold phosphate-buffered saline. Cells were suspended in H_2_O in the presence of carrier nonradioactive hemoglobin and lysed by multiple freeze-thaw cycles in dry ice-methanol baths. Globin chains were separated by high performance liquid chromatography (HPLC) with a LiChroCART 250-4 column (VWR) using gradients of acetonitrile and methanol as described [28]. Fractions were collected and radioactivity in sample analyzed using a Packard Tricarb 1600TR liquid scintillation counter.

Levels of α- γ- and β-globin mRNA were determined by RT-PCR as previously described [27]. RNA was isolated from cells using the RNeasy Mini Kit (Qiagen) according to manufacturer’s instructions. Samples were treated with DNase I and cDNA was prepared using a commercial kit (Fermentas). Custom designed primer probe sets [27] for real time PCR analysis of baboon cDNA were obtained from Applied Biosystems. Absolute numbers of globin transcripts were determined by extrapolation from standard curves prepared from the cloned amplicons. Results are expressed as a ratio of γ/γ+β mRNA.

### Bisulfite Sequence Analysis

DNA methylation of 5 CpG sites within the 5′ γ-globin promoter region was analyzed by bisulfite sequencing of DNA isolated from purified erythroid cells as previously described [24]. Genomic DNA was extracted using QIAmp DNA Blood Mini Kits (Qiagen). Bisulfite conversion was performed using Epitect Bisulfite kits (Qiagen) and the PCR amplification of the converted product performed as described [24]. PCR products were cloned in the pCR4 vector. Sequence analysis of minilysate DNA prepared from isolated clones was performed at the University of Illinois at Chicago DNA Services Facility.

### Flow Cytometry

Flow cytometry was performed to analyze the expression of CD36 and the baboon red blood cell-specific antigen (bRBC) on varying days of culture. For analysis, cells were incubated 60 minutes in phosphate-buffered saline containing 30% normal rabbit serum in the presence of fluorescein-labelled anti-CD36 (Clone FA6.152, Beckman-Coulter) and phycoerythrin-labelled anti-bRBC (BD Pharmingen) antibodies. Cells were analyzed using a Beckman-Coulter Cytomics FC500 flow cytometer.

### Statistical Significance

Statistical significance between samples was assessed using the Student’s T test.

## Results

**Figure 1:**
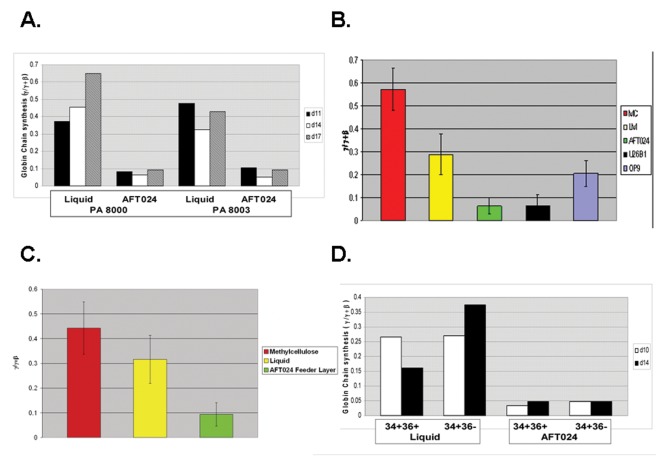
Effect of stromal cell line co-cultures on γ-globin expression during erythroid differentiation of CD34+ bone marrow progenitors. **A.** Comparison of globin chain synthesis on varying days in baboon BM-derived erythroid progenitors grown in liquid media and in AFT024 stromal cell line co-cultures. **B.** Globin chain synthesis in baboon BM-derived erythroid progenitors grown in methylcellulose media (MC), liquid media (LM), and co-cultures containing the AFT024, U26B1, or OP9 stromal cell lines. **C.** Comparison of globin mRNA expression (γ/γ+β) in baboon BM-derived erythroid progenitors grown in methylcellulose media, liquid media, or AFT024 stromal cell line co-cultures. **D.** Globin chain synthesis in CD34+ baboon BM subpopulations differing in CD36 expression.

Initial experiments to investigate the effect of fetal liver stromal cells on globin gene expression were performed by measuring globin chain synthesis during erythroid differentiation of CD34+ adult bone marrow progenitors grown in liquid media and in AFT024 co-cultures on days 11, 14, and 17 of culture. On all days, γ/γ+β synthetic ratios were significantly elevated in LM compared to AFT024 co-cultures (Figure 1A). In other experiments analysis of globin chain synthesis was performed on d14.

To extend these observations, experiments (n = 10) were conducted using CD34+ progenitors isolated from six different adult baboons, comparing γ/γ+β synthetic chain ratios and globin gene mRNA levels during erythroid differentiation in methylcellulose media, liquid media, and AFT024 co-cultures (Figure 1B). The highest levels of γ-globin chain synthesis were observed in methylcellulose media (0.57±.09 γ/γ+β). Elevated levels of γ-globin chain synthesis were also observed in liquid media (0.29±.09 γ/γ+β), but were significantly lower than in methylcellulose (p<.0001). Erythroid cells in AFT024 co-cultures expressed low, physiological levels of γ-globin (0.06±.04 γ/γ+β) that were significantly different than in methylcellulose and liquid media (p<.0001). No significant difference in the α/γ+β chain ratios between liquid (1.00.09), methylcellulose (0.97.08), and feeder layer co-cultures (1.05.09) was observed. By RT-PCR analysis, differences in γ-globin expression in liquid media, methylcellulose, and in AFT024 co-cultures were consistent with differences in globin chain synthesis (Figure 1C).

Additional co-cultures consisting of U26B1 AGM-derived and OP9 adult bone marrow-derived stromal cell lines were tested to determine whether the effect on γ-globin expression was limited to fetal liver-derived stromal cells (Figure1B). Results showed γ-globin chain synthesis during erythroid differentiation in U26B1 co-cultures was low (.064±.05 γ/γ+β) and similar to the level in AFT024 co-cultures (.064±.04 γ/γ+β) and significantly different than the elevated levels observed in liquid media (p<.0028). The γ/γ+β chain ratio during erythroid differentiation in OP9 co-cultures (0.204±.055 γ/γ+β) was not significantly different than in liquid media (.29±.09 γ/γ+β) but was significantly different than in AFT024 (p<.001) and U26B1 co-cultures (p<.05). The effect of these three stromal lines on γ-globin expression thus does not correlate with the pattern of globin gene expression characteristic of the developmental stage of origin of the stromal cell lines.

**Figure 2:**
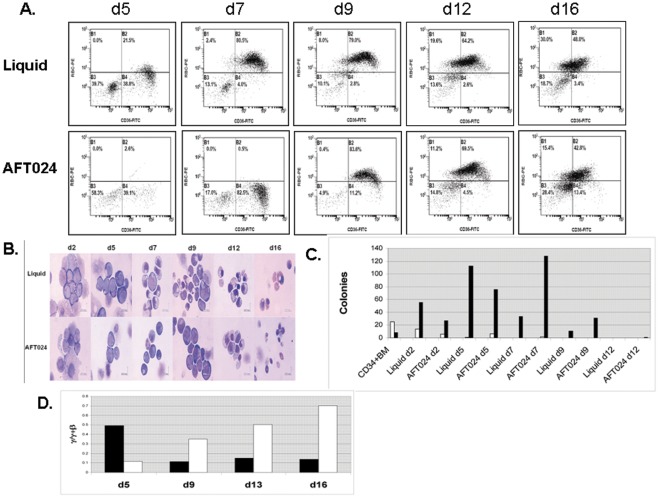
Comparison of the kinetics of erythroid differentiation of baboon CD34+ BM-derived erythroid progenitors grown in liquid media and in co-cultures with the AFT024 stromal cell line. **A.** FACS analysis of cells expressing the CD36 and bRBC antigens on varying days of culture. **B.** Analysis of cell morphology in Wright’s stained cytospin preparations on varying days of culture. **C.** Results of colony assays perfomed on varying days of culture. BFUe = white bar, CFUe = black bar. **D.** Globin mRNA expression (γ/γ+β) on varying days of culture. AFT024 co-cultures = black bar, Liquid media = white bar.

To test whether decreased γ-globin chain expression in AFT024 co-cultures was secondary to preferential expansion of more differentiated progenitors less capable of γ-globin expression, bone marrow cells were fractionated by FACS into early CD34+CD36- and late CD34+CD36+ subpopulations prior to growth in liquid media and AFT024 co-cultures. The γ/γ+β synthetic ratio was similarly reduced in both subpopulations in AFT024 co-cultures suggesting that preferential expansion of these subpopulations was not the cause of decreased γ-globin expression (Figure 1D). To further investigate whether differences in globin gene expression might be a consequence of an effect of the stromal cell line on the rate of erythroid differentiation, CD34+ BM cells were cultured in liquid media and in AFT024 co-cultures. On varying days of culture, flow cytometry was performed to analyze expression of CD36 and the bRBC antigen, cell morphology was evaluated by examination of Wright’s stained cytospin preparations, and colony assays were performed to determine numbers of BFUe and CFUe present. Results of these studies (Figure 2) showed an accelerated rate of differentiation in liquid cultures relative to AFT024 co-cultures. On d7, 80.5% of cells in liquid media were CD36+bRBC+ while those 82.5% were CD36+bRBC- in AFT024 co-culures. By d9 83.0% of cells in AFT024 co-cultures were CD36+bRBC+ suggesting that the rate of differentiation in liquid culures was accelerated 1–2 days relative to AFT024 co-cultures (Figure 2A). Examination of Wright’ stained cytospin preparation were consistent with an accelerated rate of differentiation in liquid cultures by the observation of polychromatic cells in liquid cultures and their absence in AFT024 co-cultures on d7 (Figure 2B). Furthermore, colony assays showed that that the highest numbers of CFUe were present on d5 in liquid cultures but on d7 in AFT024 co-cultures, thus providing additional evidence confirming an accelerated rate of differentiation in liquid cultures (Figure 2C). In addition to the difference in the rate of differentiation between liquid and AFT024 co-cultures, a clear difference in the pattern of globin gene expression was also observed (Figure 2D). RT-PCR analysis of γ- and β-globin transcipts on varying days of culture in liquid media showed that the fraction of γ-globin mRNA increased continuously. In contrast, cells cultured in AFT024 co-cultures exhibited evidence of a maturational switch in the pattern of globin gene expression with a high proportion of γ/γ+β expression early (d5) with a clear switch to lower γ/γ+β expression at later times in culture.

**Figure 3:**
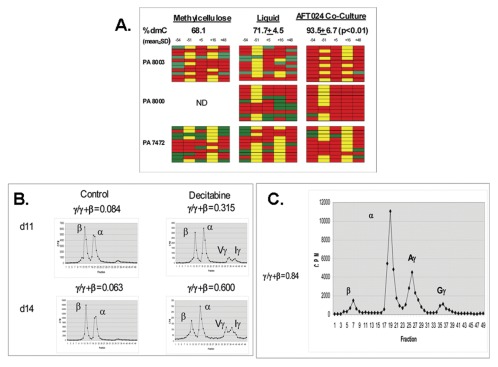
Roles of DNA methylation and development in modulation of γ-globin expression in AFT024 co-cultures. A. Bisulfite sequence analysis of DNA methylation of the γ-globin 5′ promoter region in erythroid progenitors grown in methylcellulose media, liquid media, or in co-cultures containing the AFT024 stromal cell line. Five CpG sites numbered relative to the transcription start site were analyzed. Each row represents the sequence analysis of a single amplicon cloned within the pCR4 vector. Unmethylated sites (green), methylated site (red), polymorophic sites where no CpG is present (yellow). **B.** Effect of decitabine on globin chain synthesis (γ/γ+β) during erythroid differentiation of baboon BM progenitors in AFT024 co-cultures. Decitabine (5×10^−7^ M) was added to cultures on d7. Globin chain synthesis was analyzed on d11 and d14. **C.** Globin chain synthesis (γ/γ+β) during erythroid differentiation of human cord blood progenitors in AFT024 co-cultures.

Bisulfite sequence analysis was performed to analyze whether differences in γ-globin expression were associated with differences in DNA methylation of the γ-globin gene promoter (Figure 3A). The mean level of DNA methylation (% deoxymethylcytosine; % dmC) of the γ-globin gene promoter was higher in erythroid cells in AFT024 co-cultures (93.5±6.7% dmC) compared to either methylcellulose (mean 68.3% dmC, n = 2) or liquid media (71.7±4.5 dmC, p<.01). Differences in γ-globin expression during erythroid differentiation of progenitors in the presence and absence of stromal cell line co-cultures thus were associated with differences in DNA methylation.

To address the functional significance of increased levels of DNA methylation, progenitors in AFT024 co-cultures were treated with the DNA methyltransferase inhibitor decitabine. Decitabine (5×10^−7^ M) was added on d7 and globin chain synthesis measured on d11 and d14. Decitabine increased γ/γ+β synthetic ratio to high levels on both days (d11, 0.315 γ/γ+β; d14, 0.600 γ/γ+β) compared to untreated controls (d11,.084 γ/γ+β; d14,.063 γ/γ+β; Figure 3B). The ability of decitabine to increase γ-globin expression in cells grown in AFT024 co-cultures models the in vivo effects of this drug [12].

To determine whether γ-globin expression during erythroid differentiation of progenitors derived from an earlier developmental stage would be similarly affected by stromal cell co-cultures, human cord blood-derived CD34+ cells were isolated and grown in AFT024 co-cultures. We were unable to obtain baboon cord blood for these studies. Globin chain synthesis measured in two independent cord blood-derived cultures showed a high level of γ-globin synthesis (γ/γ+β = 0.84, 0.54, n = 2) in AFT024 co-cultures (Figure 3C). These results are thus consistent with previous studies showing that globin gene expression during adult eythropoiesis, but not the developmental globin switch, is sensitive to modulation by extrinsic factors.

**Figure 4:**
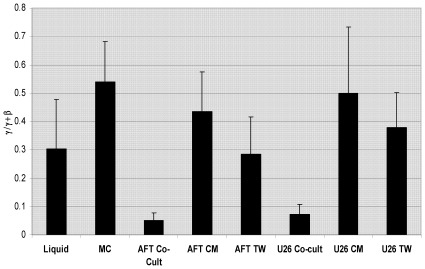
Effect of AFT024 and U26B1 cell lines on γ-globin expression requires cell-cell contact. Analysis of γ-globin chain synthesis (γ/γ+β) in baboon BM-derived CD34+ cells cultured in methylcellulose (MC), liquid media (LM), and AFT024 and U26 B1 co-cultures, transwell (TW) cultures and in LM containing conditioned media (CM) from the AFT024 and U26B1 cell lines. The single asterisk denotes statistical significance (p<.01) relative to AFT co-cultures. The double asterisk denotes statistical significance (p<.01) relative to U26B1 co-cultures.

Additional experiments were performed to determine whether the effect of stromal cell lines on γ-globin expression required cell-cell contact or was mediated by a soluble factor. Comparison of γ-globin chain synthesis in BM-derived CD34+ cells grown in methylcellulose, liquid media, AFT024 and U26B1 stromal cell co-cultures, transwell cultures, and liquid media containing 40% conditioned media derived from AFT0254 or U26B1 monolayers showed that γ-globin synthesis (γ/γ+β) was significantly increased (p<.01) in methylcellulose, (.538+.143 γ/γ+β), liquid media (.302+.175 γ/γ+β), AFT024 transwell cultures (.283+.133 γ/γ+β), U26B1 transwell cultures (.379+.120 γ/γ+β), and cultures containing conditioned media derived from either the AFT024 (.453+.138 γ/γ+β) or U26B1 cell lines (.500+.233 γ/γ+β) compared to cells grown in co-cultures with AFT024 (.051+.027 γ/γ+β) or U26B1 stromal cells (.073+.032 γ/γ+β; Figure 4). Cell growth was enhanced 3–5 fold in stromal cell co-cultures and also in the presence of 40% conditioned media compared to liquid media alone showing that differences in γ-globin expression were unrelated to differences in proliferation.

Fibronectin, an isolated component of the erythroid niche, has been reported to mediate growth and survival of erythroid progenitors [15] Therefore experiments were performed to determine whether the effect of co-culture with stromal cell lines on globin expression might be mediated by isolated extracellular matrix components. Results showed γ-globin synthesis (γ/γ+β) was not significantly different in CD34+ bone marrow cells cultured on plates coated with gelatin (.534 γ/γ+β), collagen (.557 γ/γ+β), fibronectin (.509 γ/γ+β), laminin (.601 γ/γ+β) and liquid media (.504 γ/γ+β) and was increased compared to AFT024 stromal cell co-cultures (.081 γ/γ+β) demonstrating that the effect of stromal cells was not mediated by these extracellular matrix components.

## Discussion

The baboon is considered an excellent animal model to test the in vivo activity of HbF-inducing agents because the structure and regulation of genes within the β-globin gene domain are highly conserved between baboon and man. True fetal developmental stage-specific expression of the γ-globin gene is observed only in simian primates [29] and recent results have shown that the human γ-globin gene in transgenic mice is regulated as an embryonic-stage gene, limiting the utility of the transgenic mouse model [30]. Furthermore, results in baboons have been successfully translated to human clinical trials [12]. The high levels of HbF expression we have observed in baboon erythroid progentor cells cultured in the absence of stromal cells are consistent with previous studies of globin gene expression in cultured baboon bone marrow cells [24], [31], [32] and although the baboon serves as an excellent in vivo model, this phenomenon has made the in vitro testing of HbF-inducing agents difficult to perform in this species. The use of the AFT024 stromal cell line co-culture system that results in near-physiological levels of HbF expression will allow the in vitro screening and in vivo pre-clinical testing of HbF-inducing agents to be performed within a single species that is highly similar to man.

This report provides the first data supporting the model that γ-globin gene expression during erythroid differentiation of adult-derived progenitors is subject to modulation by stromal-associated micro-environmental factors within the erythroid niche. While a soluble humoral factor was previously reported to inhibit the increased HbF synthesis in cultured human adult-derived BFUe [33], our experiments show that the mechanism responsible for the effect on HbF synthesis in these experiments requires cell-cell contact between the bone marrow cells and stromal cells rather than soluble factors. In addition, our results show that the difference in γ-globin expression between CD34+ BM cells grown in liquid media compared to AFT024 co-cultures is associated with a difference in the rate of erythroid differentiation. CD34+ BM cells grown in liquid media exhibit accelerated differentiation relative to those grown in AFT024 co-cultures. In addition, cells grown in AFT024 co-cultures show evidence of a maturational switch that appears to be lacking in liquid media. Thus the relatively rapid differentiation in liquid media may commit cells to an irreversible fetal globin expression program prior to the maturational switch necessary for predominant adult globin expression. Stromal cells are known to express growth factors capable of expanding more primitive progenitors [21]–[23] and it is possible that these factors inhibit rapid induction of differentiation and allow the maturational switch from γ- to β-globin production. Such a maturational switch has been previously reported in human BFUe grown in methylcellulose and human CD34+ progenitors grown in liquid media on fibronectin coated dishes [34], [35]., although in our experiments γ-globin expression remained high in fibronectin coated dishes. It is possible that differential sensitivity of baboon and human progenitors to growth factors may be responsible for the ability of baboon progenitors to synthesize high HbF levels relative to human progenitors in liquid cultures. Interestingly, our results show that effects of various stromal cell lines on globin gene expression in the co-culture system did not correlate with the developmental stage of origin of the stromal lines. Specifically, the yolk sac and fetal liver-derived cell lines decreased γ-globin expression while high levels of γ-globin expression were observed in co-cultures with an adult BM-derived stromal cell line. Further analysis of differential gene expression patterns between these lines could identify candidate factors responsible for these effects.

While the stromal micro-environment is capable of inhibiting the increased γ-globin expression observed in cultured adult bone marrow derived erythroid progenitors, it is incapable of repressing γ-globin expression in cord blood-derived progenitors or decitabine-treated adult bone marrow-derived progenitors, suggesting that permissive epigenetic signals such as DNA hypomethylation of the γ-globin promoter may be dominant to repressive stromal cell factors. Our results support the model stating that developmental switching is controlled by a cell-intrinsic developmental clock based on transplantation experiments perfomed in sheep [2], [3]. These transplantation experiments were performed in a single species, in contrast to transplantation experiments of human cord blood cells into NOD/SCID mice where a switch from predominately fetal to adult globin gene expression was observed [20]. Cord blood contains a mix of progenitors programmed to express either a fetal or adult globin program and it is possible that species differences in growth factors cross-reactivities and growth factor requirements of these different progenitors could have allowed preferential expansion and differentiation of progenitors expressing the adult globin program in the NOD/SCID mouse. Identification of the specific extrinsic signals produced by stromal cells that allow induction of the maturational switch in adult progenitors may foster the development of additional therapeutic strategies that interfere with this switch to enhance HbF expression for the treatment of hemoglobinopathies.

## References

[1] Stamatoyannopoulos G (2001). Molecular and Cellular basis of hemoglobin switching..

[2] Zanjani Ed, Lim G, McGlave PB, Clapp JF, Man LI (1982). Adult haematopoietic cells transplanted to sheep fetuses continue to produce adult globins.. Nature.

[3] Wood WG, Bunch C, Kelly S, Gunn Y, Breckon G (1985). Control of hemoglobin switching by a developmental clock?. Nature.

[4] Papayannopoulou T, Brice M, Stamatoyannopoulos G (1986). Analysis of human hemoglobin switching in MEL X human fetal erythroid cell hybrids.. Cell.

[5] Papayannopoulou Th, Brice M, Stamatoyannopoulos G (1976). Stimulation of fetal hemoglobin synthesis in bone marrow cultures from adult individuals. Proc Natl Acad Sci USA..

[6] Migliaccio AR, Migliaccio G, Brice M, Constantoulakis P, Stamatoyannopoulos G (1990). Influence of recombinant hematopoietins and of fetal bovine serum on the globin synthetic pattern of human BFUe.. Blood.

[7] Fujimori Y, Ogawa M, Clark SC, Dover GJ (1990). Serum-free culture of enriched hematopoietic progenitors reflects physiologic levels of fetal hemoglobin biosynthesis.. Blood.

[8] Peschle C, Gabbianelli M, Testa U, Pelosi E, Barberi T (1993). c-kit ligand reactivates fetal hemoglobin synthesis in serum-free culture of stringently purified normal adult burst-forming unit-erythroid.. Blood.

[9] Bhanu NV, Trice TA, Lee YT, Gantt NM, Oneal P (2005). A sustained and pancellular reversal of gamma-globin gene silencing in adult human eryhroid precursor cells.. Blood.

[10] Al-Khatti A, Veith RW, Papayannopoulou Th, Fritsch EF, Goldwasser E (1987). Stimulation of fetal hemoglobin synthesis by erythropoietin in baboons. N Engl J Med..

[11] Lavelle D, Molokie R, Ducksworth J, DeSimone J (2001). Effects of hydroxyurea, stem cell factor, and erythropoietin in combination on fetal hemoglobin in the baboon.. Exp Hematol.

[12] Saunthararajah Y, Lavelle D, DeSimone J (2004). DNA hypomethylating agents and sickle cell disease.. Br J Hematol.

[13] Atweh G, Fathallah H (2010). Pharmacologic induction of fetal hemoglobin production.. Hematol Oncol Clin North Am.

[14] Chasis J, Mohandas N (2008). Erythroblastic islands: niches for erythropoiesis.. Blood.

[15] Eshghi S, Vogelezang MG, Hynes RO, Griffin LG, Lodish HF (2007). Alpha4beta1 integrin and erythropoietin mediate temporally distinct steps in erythropoiesis: integrins in red cell development.. J Cell Biol.

[16] Dev A, Fang J, Sathyanarayana P, Pradeep A, Emerson C (2010). During EPO or anemia challenge, erythroid progenitor cells transit through a selectively expandable proerythroblast pool.. Blood.

[17] Qiu C, Olivier EN, Velho M, Bouhassira EE (2008). Globin switches in yolk sac-like primitive and fetal-like definitive red blood cells produced from human embryonic stem cells.. Blood.

[18] Lee KY, Fong BSP, Tsang KS, Lau TK, Ng PC (2011). Fetal stromal niches enhance human embryonic stem cell-derived hematopoietic differentiation and globin switch.. Stem Cells Dev.

[19] Chang CJ, Mitra K, Koya M, Velho M, Desprat R (2011). Production of embryonic and fetal-like red blood cells from human induced pluripotent stem cells.. PLoS One.

[20] Neildez-Nguyen TMA, Wajcman H, Marden MC, Bensidhoun M, Moncolin V (2002). Human eryrthroid cells produced ex vivo at large scale differentiate into red blood cells in vivo.. Nature Biotech.

[21] Moore KA, Ema H, Lemischka IR (1991). In vitro maintenance of highly purified, transplantable hematopoietic stem cells.. Blood.

[22] Nakano T, Kodama H, Honjo T (1994). Generation of lymphohematopoietic cells from embryonic stem cells in cultures.. Science.

[23] Kusadasi N, Oostendorp RA, Koevoet WJ, Dzierzak EA, Ploemacher RE (2002). Stromal cells from the murine embryonic aorta-gonad-mesonephros region, liver and gut mesentery expand human umbilical cord blood-derived CAFC (week6) in extended long-term cultures.. Leuk.

[24] Chin J, Singh M, Banzon V, Vaitkus K, Ibanez V (2009). Transcriptional activation of the γ-globin gene n baboons treated with decitabine and in cultured erythroid progenitor cells involves different mechanisms. Exp Hematol..

[25] Araki H, Mahmud N, Milhem M, Nunez R, Xu M (2006). Expansion of human umbilical cord blood SCD-repopulating cells using chromatin-modifying agents.. Exp Hematol.

[26] Singh M, Lavelle D, Vaitkus K, Mahmud N, Hankewych M (2007). The γ-globin gene promoter progressively demethylates as the hematopoietic stem progenitor cells differentiate along the erythroid lineage in baboon fetal liver and adult bone marrow.. Exp Hematol.

[27] Banzon V, Ibanez V, Vaitkus K, Ruiz MA Peterson K (2011). siDNMT1 increases γ-globin expression in chemical inducer of dimerization (CID)-dependent mouse βYAC bone marrow cells and in baboon erythroid progenitor cell cultures.. Exp Hematol.

[28] Leone L, Monteleone M, Gabutti V, Amione C (1985). Reversed phase high performance liquid chromatography of human hemoglobin chains.. J Chromatog.

[29] Tagle DA, Koop BF, Goodman M, Slightom JL, Hess DL (1988). Embryonic epsilon and gamma globin genes of a prosimian primate (Galago crassicaudatus). Nucleotide and amino acid sequences, developmental regulation, and phylogenetic footprints.. J Mol Biol.

[30] Sankaran VG, Xu J, Ragoczy T, Ipppolito GC, Walkley CR (2009). Developmental and species-divergent globin switching are driven by BCL11A.. Nature.

[31] DeSimone J, Heller P, Adams JG (1979). Hemopoietic stress and fetal hemoglobin synthesis: comparative studies in vivo and in vitro.. Blood.

[32] Torrealba de Ron A, Papayannpoulou Th, Stamatoyannopoulos G (1985). Studies of HbF in adult nonanemic baboons: HbF expression in erythroid colonies decreases as the level of maturation of erythroid progenitors advances.. Exp Hematol.

[33] Papayannopoulou Th, Kurachi S, Nakamoto B, Zanjani ED, Stamatoyannopoulos G (1982). Hemoglobin switching in culture: evidence for a humoral factor that induces switching in adult and neonatal, but not fetal erythroid cells.. Proc Natl Acad Sci USA.

[34] Papayannopoulou Th, Kurachi S, Brice N, Nakamoto B (1981). Asynchronous synthesis of HbF and HbA during erythroblast maturation. II Studies of Gγ, Aγ, and β chain synthesis in individual clones from neonatal and adult BFU-E cultures.. Blood.

[35] Ni H, Yang X-D, Stoeckert C (1999). Maturation and developmental stage-related changes in fetal globin gene expression are reproduced in transiently transfected primary adult erythroblasts.. Exp Hematol.

